# Amyloid‐Templated Palladium Nanoparticles for Water Purification by Electroreduction

**DOI:** 10.1002/anie.202116634

**Published:** 2022-01-31

**Authors:** Jie Teng, Mohammad Peydayesh, Jiandong Lu, Jiangtao Zhou, Peter Benedek, Robin E. Schäublin, Shijie You, Raffaele Mezzenga

**Affiliations:** ^1^ State Key Laboratory of Urban Water Resource and Environment School of Environment Harbin Institute of Technology No. 73, Huanghe Road Nangang District, Harbin 150090 P. R. China; ^2^ Department of Health Sciences & Technology ETH Zurich Schmelzbergstrasse 9 8092 Zurich Switzerland; ^3^ Department of Information Technology and Electrical Engineering ETH Zurich 8092 Zurich Switzerland; ^4^ Scientific Center for Optical and Electron Microscopy (ScopeM) ETH Zurich Otto-Stern-Weg 3 8093 Zurich Switzerland; ^5^ Department of Materials ETH Zurich Wolfgang Pauli Strasse 10 8093 Zurich Switzerland

**Keywords:** Amyloid Fibrils, Electroreduction, Nanocatalysts, Palladium, Water Purification

## Abstract

Electrocatalysis offers great promise for water purification but is limited by low active area and high uncontrollability of electrocatalysts. To overcome these constraints, we propose hybrid bulk electrodes by synthesizing and binding a Pd nanocatalyst (nano‐Pd) to the electrodes via amyloid fibrils (AFs). The AFs template is effective for controlling the nucleation, growth, and assembly of nano‐Pd on the electrode. In addition, the three‐dimensional hierarchically porous nanostructure of AFs is beneficial for loading high‐density nano‐Pd with a large active area. The novel hybrid cathodes exhibit superior electroreduction performance for the detoxification of hexavalent chromium (Cr^6+^), 4‐chlorophenol, and trichloroacetic acid in wastewater and drinking water. This study provides a proof‐of‐concept design of an AFs‐templated nano‐Pd‐based hybrid electrode, which constitutes a paradigm shift in electrocatalytic water purification, and broadens the horizon of its potential engineered applications.

## Graphical abstract

A high‐density palladium nanocatalyst is successfully immobilized on bulk electrodes via a 3D‐structured amyloid fibrils template. The novel hybrid electrodes exhibit superior electroreduction performance for detoxification of wastewater and drinking water.

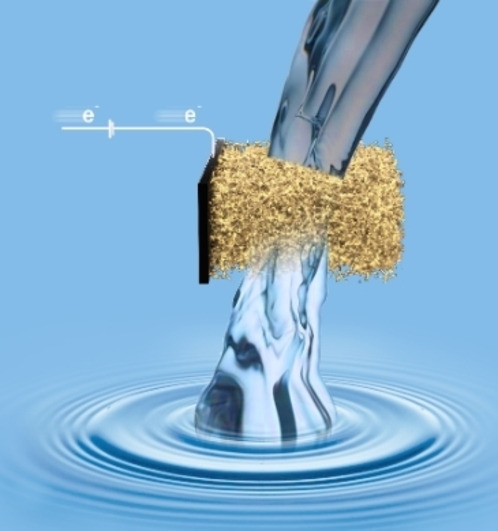

Water contamination caused by highly toxic pollutants is producing devastating impacts on worldwide public health and ecosystems.^[1]^ Transforming the contaminants into less toxic or nontoxic products by destructive technologies has become a critical strategy at different stages of water and wastewater treatment.^[2]^ Electrochemistry has been widely advocated as a prospective technique for decentralized water purification due to its environmental friendliness, operational simplicity, and high energy efficiency.^[3]^ Nevertheless, an electrode's activity, practicability, and sustainability remain major challenges for the electrochemical removal of pollutants in water.

Electroreduction using palladium‐based electrodes has been extensively investigated as a promising approach to treat a variety of contaminants, including hexavalent chromium (Cr^6+^), nitrate (NO_3_^−^), and chlorophenols.^[4]^ Remarkably, nanometer‐sized palladium (nano‐Pd) is highly stable with considerable Fermi potential, and can activate the adsorbed H_2_ into the highly‐active atomic hydrogen (H*), making it an efficient electroreduction catalyst.^[5]^ Moreover, nano‐Pd has a much higher surface‐to‐volume ratio than bulk materials, which contributes to a more significant number of catalytically‐active sites for lowering kinetic barriers and promoting reaction efficiency.^[6]^ Yet, practical applications face the constraints of high cost, complexity of preparation, and dispersion of uncontrollable nano‐Pd in an aqueous solution. To address these issues, much effort has been devoted to stabilizing a nanocatalyst on bulk substrates. However, loading capacity, dispersivity, and catalytic activity are markedly impeded by the two‐dimensional (2D) surface area of the bulk substrates.^[7]^ Therefore, developing bulk substrate‐based nano‐Pd with high density and high catalytic activity is highly desirable in a cost‐effective and easy‐to‐handle pathway.

The self‐assembled proteins have offered an outstanding method for the synthesis of nanoparticles (e.g., Au, Pd, Ag, and Fe) with unique functions, which is attributable to the presence of multiple specific functional groups.^[8]^ Particularly, the milk‐based β‐lactoglobulin (BLG) protein extracted from the inexpensive and nontoxic byproducts of dairy factories can efficiently generate amyloid fibrils (AFs) with a representative cross β‐sheet structure.

For water purification applications, AFs‐templated metallic nanocatalysts on various membranes can be used as adsorbents and/or catalysts for improved decontamination efficiency.^[9]^ Recent literature has also demonstrated that AFs could serve as adhesive decoration carriers with various shapes, compositions, and structures.^[10]^ These carriers, with excellent chemical robustness and genetically programmable functionality, have been successfully used on substrates, including silica, titanium, and carbon‐based materials. AFs also have advantages, including sustainability, good mechanical strength, and adhesiveness.^[11]^ Besides that, the 3D‐structured AFs enable higher Pd loading and a larger electrochemically active surface area (ECSA). Based on these characteristics, improved electroreduction performance by AFs‐templated nano‐Pd (AFs‐Pd) is highly anticipated.

Herein, with the aim of nano‐Pd stabilization for electroreduction water purification, we use AFs as the template to synthesize nano‐Pd in situ on carbon paper (CP‐AFs‐Pd) and titanium suboxide reactive electrochemical membrane (TiSO‐REM‐AFs‐Pd). CP‐AFs‐Pd and TiSO‐REM‐AFs‐Pd were investigated in batch and flow‐through electroreduction systems to detoxify typical toxic contaminants, i.e., Cr_2_O_7_^2−^/CrO_4_^2−^ (Cr^6+^), 4‐chlorophenol (4‐CP), and trichloroacetic acid (TCAA).

The preparation of the CP‐AFs‐Pd hybrid cathode is illustrated in Figure 1 (see details in Supporting Information) according to our previous studies.^[12]^ As shown in scanning electron microscopy (SEM) images in Figures 2a and S1a, AFs were uniformly formed on carbon paper with AFs diameters ranging between 5 nm and 10 nm. The dense packing of nano‐Pd on AFs increased the diameters further to 8–15 nm. The three‐dimensional (3D) porous nanostructure of AFs enabled larger nano‐Pd loadings, providing a larger catalytically active area. An in‐depth examination of the binding between nano‐Pd and AFs by Fourier transform infrared spectroscopy (FTIR) revealed peaks at 1250 cm^−1^, 1530 cm^−1^, and 1663 cm^−1^ for amide I, amide II, and amide III, respectively, suggesting the characteristic signatures of AFs (Figure 2b).^[13]^ These results confirmed the high‐density cross‐linkage of nano‐Pd and the CP surface bridged by AFs.

**Figure 1:**
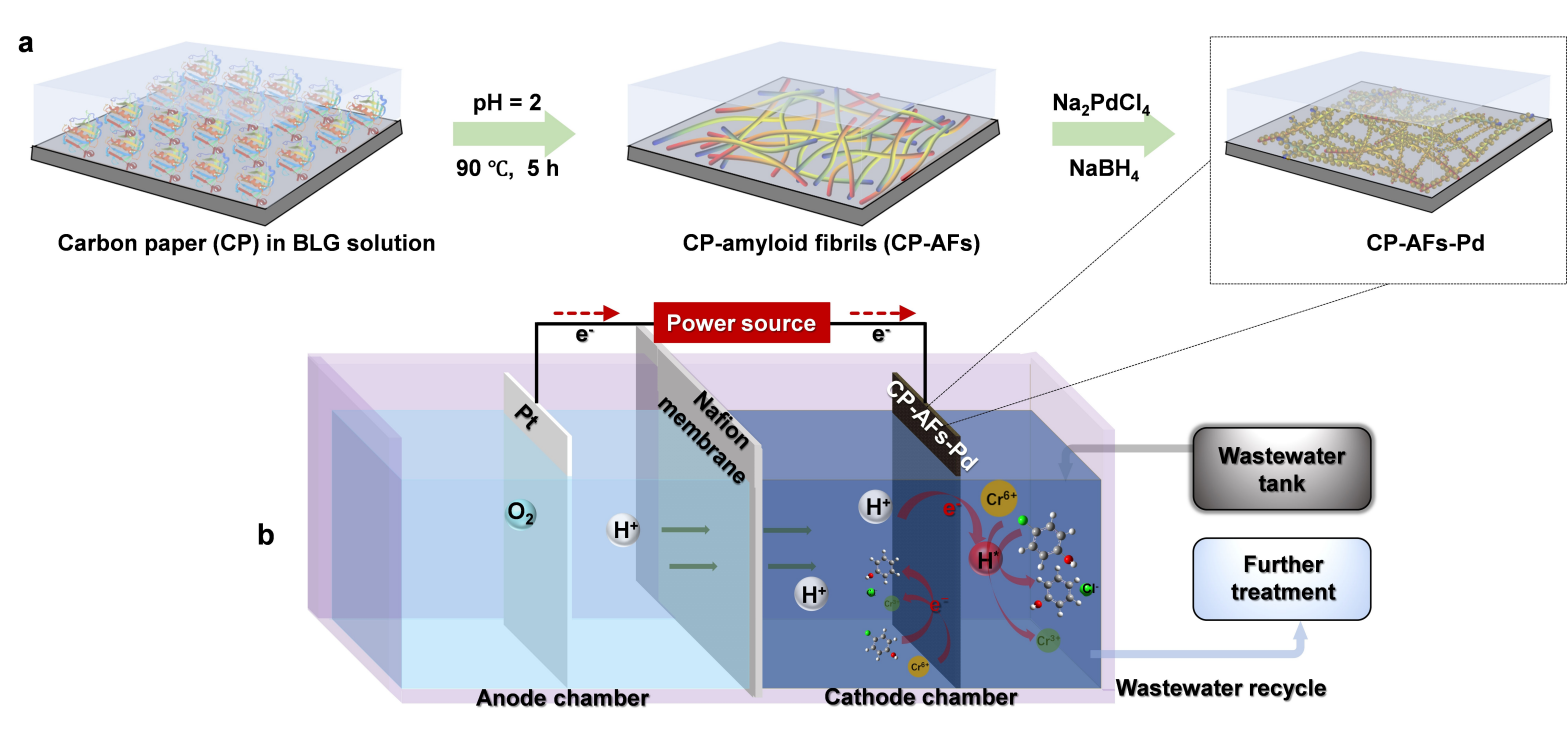
Schematic diagram of a) the fabrication process of CP‐AFs‐Pd and b) the setup for electroreduction water purification.

**Figure 2:**
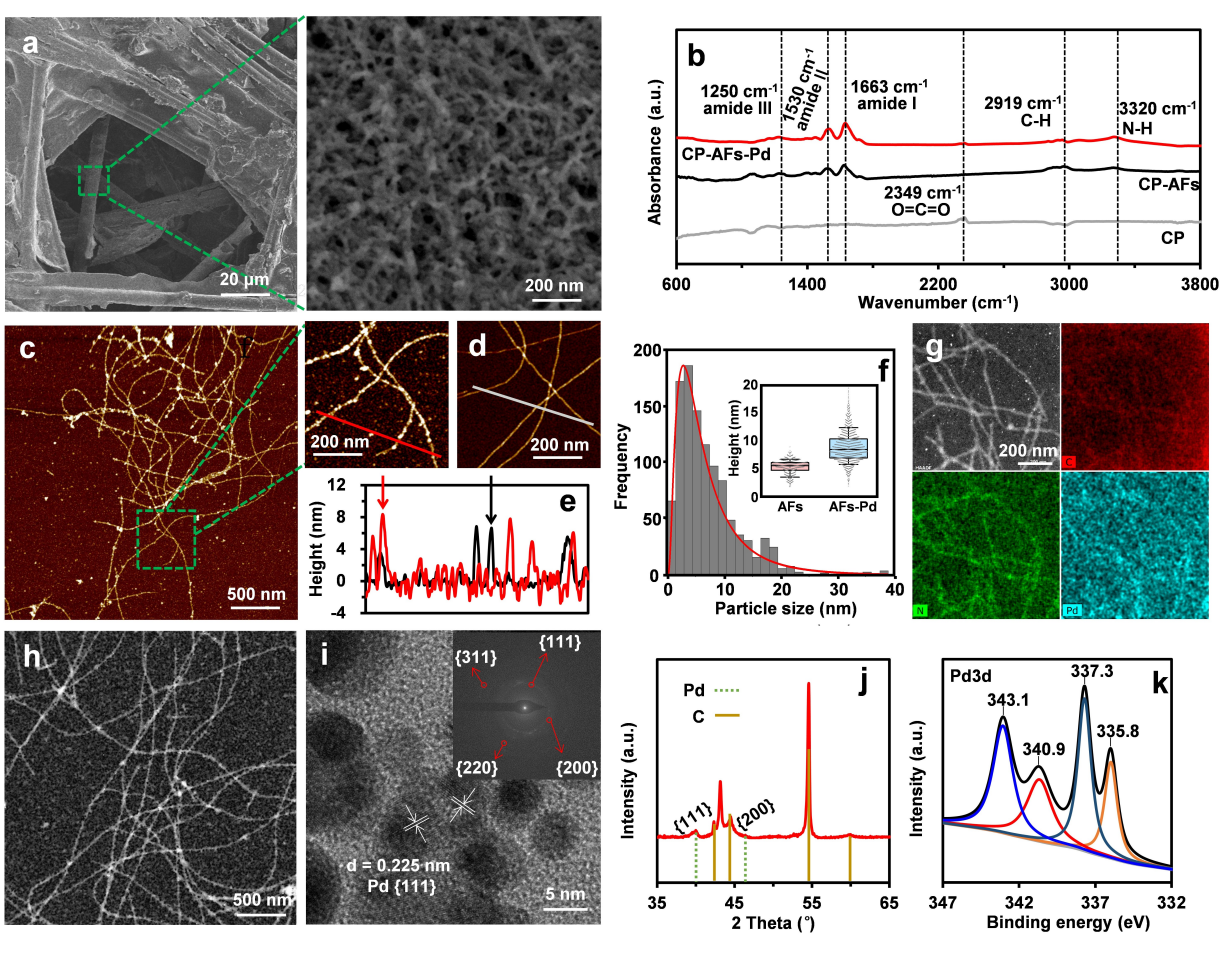
Characterization of the CP‐AFs‐Pd cathode. a) SEM images of the CP‐AFs‐Pd surface at two magnifications. b) FTIR spectra of CP, CP‐AFs, and CP‐AFs‐Pd. AFM images of c) AFs‐Pd and d) AFs on the mica surface. e) Height profiles of the red and white auxiliary lines in the AFM images of AFs‐Pd and AFs, respectively. f) Height distribution of nano‐Pd (inset showing height distribution of AFs and AFs‐Pd). g) STEM‐EDX chemical maps and h) TEM image of AFs‐Pd nanocomposites. i) HRTEM image of nano‐Pd coated on AFs, with the inset showing the SAED pattern. j) XRD patterns of CP‐AFs‐Pd, which refer to characteristic peaks of Pd (JCPDS 46‐1043) and C (JCPDS 26‐1076). k) XPS spectra of CP‐AFs‐Pd.

The atomic force microscopy (AFM) images show the AFs‐Pd composites, which are polydisperse, with an average contour length of several microns and a diameter of less than 10 nm (Figure 2c). The reduced nano‐Pd was distributed uniformly on the surface of AFs, increasing the average height from 5 nm to 8 nm (Figures 2d, e). The height distribution of AFs and AFs‐Pd is also shown in Figure 2f. Moreover, the energy‐dispersive X‐ray spectroscopy (EDX) mapping performed in scanning transmission electron microscopy (STEM) of AFs‐Pd revealed that the distribution of Pd coincided with that of C and N involved in amyloid, which confirmed the essential role of AFs as a bridging template for nano‐Pd formation (Figure 2g).

Transmission electron microscopy (TEM) images (Figures 2h, i) illustrate the crystalline structure of nano‐Pd with a diameter of approximately 5 nm, which is in accordance with AFM observation. The crystallization to a relatively high degree was confirmed by the selected area electron diffraction (SAED) pattern exhibiting spots corresponding to {111}, {200}, {220}, and {311} lattice planes (Figure 2i inset). Continuous lattice fringes with a spacing of 0.225 nm were observed for nano‐Pd, which was also consistent with the {111} plane (Figure 2i) and X‐ray diffraction (XRD) data (JCPDS 46‐1043, Figure 2j).^[14]^ Following the coating of nano‐Pd on CP‐AFs, the diffraction peaks observed at 2*θ*=40.7° and 47.2° fit well with standard peaks of the face‐centered cubic crystallographic structure of metallic Pd being assigned to {111} and {200} reflections, respectively. To further confirm the formation of nano‐Pd, X‐ray photoelectron spectroscopy (XPS) characterization of CP‐AFs‐Pd was also performed. Figure 2k shows the Pd 3d spectra with two characteristic peaks at binding energies of 335.8 eV and 340.9 eV, corresponding to Pd 3d_5/2_ (335.0 eV) and Pd 3d_3/2_ (340.3 eV), respectively.^[15]^ This indicates the presence of Pd^0^, and the slight shift may be the consequence of the interaction between nano‐Pd and AFs.

The electroreduction water purification by the CP‐AFs‐Pd hybrid electrode was evaluated by examining Cr^6+^ and 4‐CP removal upon batch tests (Figure 1b). As shown in Figures 3a, b, Pd‐free CP and CP‐AFs exhibited insignificant removal of both pollutants (<5 %). Remarkably, CP‐AFs‐Pd achieved efficient electroreduction of Cr^6+^ and 4‐CP with removal efficiency as high as 92 % and 98 %, respectively, at −1.2 V versus standard hydrogen electrode (vs. SHE) within 40 min. These results demonstrated the nano‐Pd to be responsible for electroreduction of Cr^6+^ and 4‐CP, as also evidenced by additional data in Figures S2, S3, and S4. Moreover, two interfacial mechanisms relevant to the electroreduction process, i.e., H*‐mediated indirect reduction and direct electron transfer, were confirmed by H*‐scavenging tests using tertiary butanol (TBA, 10 mM; Figure S5) and cyclic voltammetry (CV; Figure S6).

**Figure 3:**
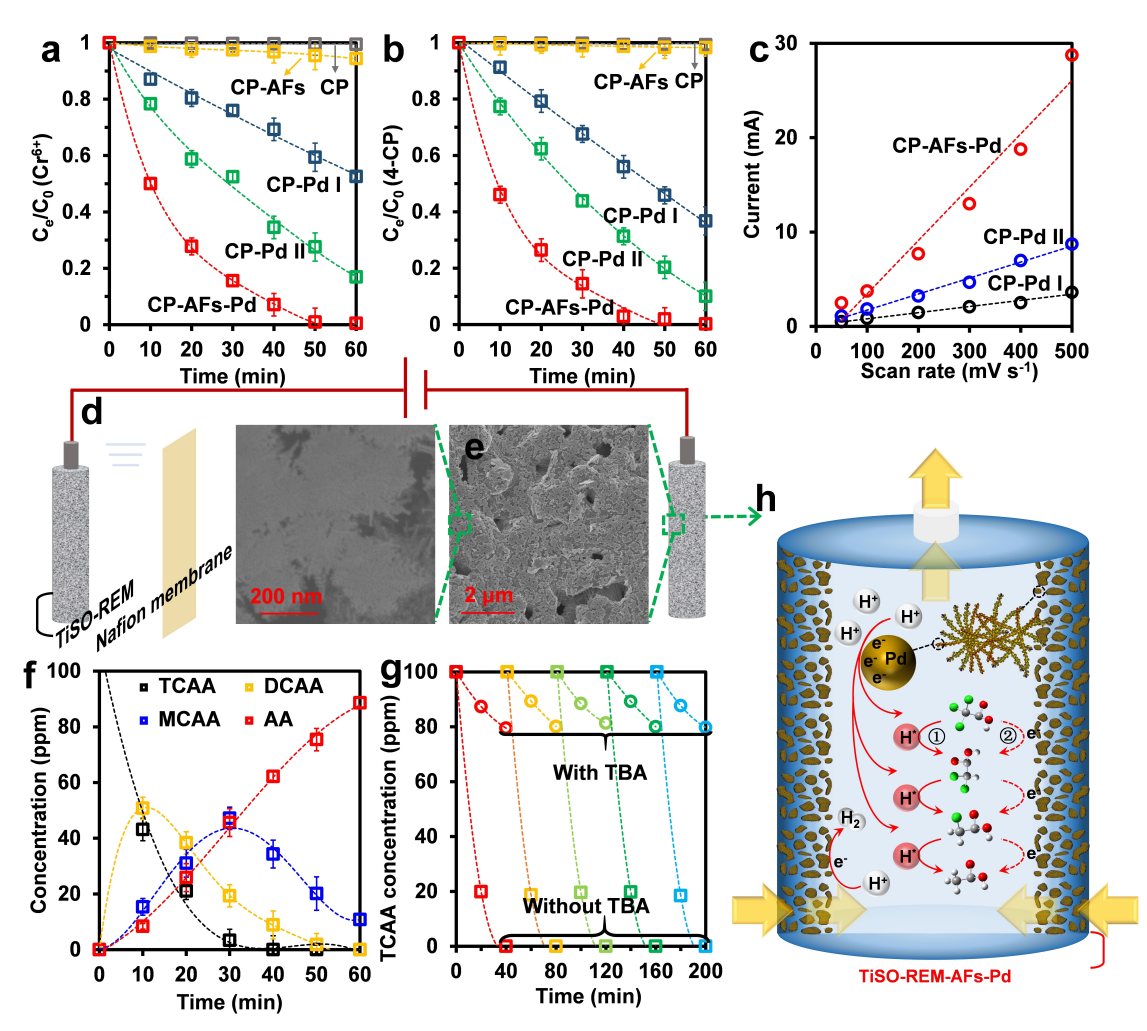
Electroreduction of a) Cr^6+^ and b) 4‐CP by various cathodes. c) Double‐layer current (0.8 V vs. SHE) vs. scan rate based on the CV curves in Figure S9. d) Schematic diagram of the experimental flow‐through reactor. e) SEM images of TiSO‐REM‐AFs‐Pd at two magnifications. f) Concentration variation of the intermediates and the final product during the electroreduction of TCAA by TiSO‐REM‐AFs‐Pd. g) Five‐cycle electroreduction of TCAA with and without TBA by TiSO‐REM‐AFs‐Pd. h) Schematic illustration of the mechanism for the electroreduction of TCAA. Initial TCAA concentration=100 ppm, pH=6.8, supporting electrolyte concentration=5 mM, applied voltage=−1.2 V vs. SHE.

To confirm the essential role of AFs in the electroreduction process, two commercially available AFs‐free cathodes (20 mm×20 mm) were also evaluated, i.e., CP‐Pd I and CP‐Pd II (Figure S8). According to the inductively coupled plasma optical emission spectrometry (ICP‐OES), the Pd loading contents of CP‐Pd I, CP‐Pd II, and CP‐AFs‐Pd were 3.1 μg cm^−2^, 360 μg cm^−2^, and 200 μg cm^−2^, respectively. Despite a lower Pd content than CP‐Pd II, CP‐AFs‐Pd exhibited significantly higher electroreduction efficiency than the AFs‐free cathodes (Figures 3a, b). The superiority of CP‐AFs‐Pd was very likely the result of the participation of AFs in the nano‐Pd formation. Based on Figures S9 and 3c, ECSA of CP‐AFs‐Pd was calculated to be 2457 cm^2^, which was higher than that of both CP‐Pd I (283 cm^2^) and CP‐Pd II (739 cm^2^). Therefore, the 3D‐structured AFs (as in CP‐AFs‐Pd) offered many more sites for nano‐Pd growth than the 2D surface of CP (as in CP‐Pd), thus contributing to a larger Pd surface area for the electroreduction reaction.

To further explore the universal feasibility of AFs, we investigated TiSO‐REM‐AFs‐Pd for more practical water purification applications.^[16]^ The TiSO electrode exhibited a hollow porous structure that allowed flow‐through operation (Figures 3d, h). Figure 3e illustrates the uniform coating of nano‐Pd on the flower‐like structured TiSO electrode surface bridged by AFs.

Moreover, the 3D porous architecture created by AFs provided the electrode with hierarchical channels. The micro‐/meso‐scale pores offered active sites for electroreduction reactions, while the macro‐scale pores facilitated mass transport by enhanced convection. We next examined the performance of TiSO‐REM‐AFs‐Pd for detoxifying TCAA, which is a trace refractory chlorinated organic pollutant that is frequently detected in water supply systems.^[17]^

The TiSO‐REM‐AFs‐Pd cathode exhibited excellent TCAA reduction, approaching 100 % within 40 min (Figure 3f). TCAA was eventually electroreduced to acetic acid (AA), with dichloroacetic acid (DCAA) and monochloroacetic acid (MCAA) as the main intermediate products (Figures 3f, h). These intermediates could be mineralized more easily by subsequent oxidative degradation. Adding TBA again led to a 78 % decrease in the reduction of TCAA (Figure 3g), illustrating the essential contribution of H* to electroreduction. The reusability and the stability of nano‐Pd in the flow‐through electroreduction system were also assessed. After 5 cycles of tests, high reduction efficiency could be sustained with a negligible change of surface morphology of the cathode, and an insignificant amount of Pd element (<0.02 ppm) was detected in the solution (Figures 3g and S10). This indicated the relatively high stability of AFs‐Pd on TiSO‐REM.

In summary, we have developed a new bio‐templated cathode by immobilizing nano‐Pd on the substrate electrode surface through AFs for electroreduction of pollutants in wastewater and drinking water. The unique 3D structure and multiple functional groups of AFs enabled the formation of high‐density nano‐Pd with a diameter of approximately 5 nm, which exhibited extraordinary electroreduction performance in converting Cr^6+^ and 4‐CP into Cr^3+^ and phenol, respectively. The electroreduction performance of the AFs‐Pd cathode was further validated in the flow‐through reactor by the detoxification of pollutants from the water supply system. These unique reduction performances were derived from the effective stabilization of H* by the novel cathodes. Compared with previous studies, excellent and versatile electroreduction performance was achieved in the current work (Table S1). Furthermore, the energy consumption was calculated to be 0.12 kWh m^−3^ and 0.11 kWh m^−3^ for complete reduction of Cr^6+^ and 4‐CP, respectively, in CP‐AFs‐Pd system, and 0.05 kWh m^−3^ for complete reduction of TCAA in TiSO‐REM‐AFs‐Pd system. These values are much lower than those commonly reported for advanced oxidation/reduction processes.^[18]^ More importantly, the easy production of AFs from whey protein highlights the affordable nature of cathode materials and markedly lowers the total cost of the electroreduction process. Therefore, this technology could be significantly valuable for addressing the pressing problem of removing refractory pollutants in water purification.

## Conflict of interest

The authors declare no conflict of interest.

## Supporting information

As a service to our authors and readers, this journal provides supporting information supplied by the authors. Such materials are peer reviewed and may be re‐organized for online delivery, but are not copy‐edited or typeset. Technical support issues arising from supporting information (other than missing files) should be addressed to the authors.

Supporting Information

## Data Availability

The data that support the findings of this study are available from the corresponding author upon reasonable request.

## References

[1a] M. Peydayesh , R. Mezzenga , Nat. Commun. 2021, 12, 3248; 34059677 10.1038/s41467-021-23388-2 PMC8166862

[1b] F. Zhao , M. Peydayesh , Y. Ying , R. Mezzenga , J. Ping , ACS Appl. Mater. Interfaces 2020, 12, 24521–24530. 32368892 10.1021/acsami.0c07846

[2a] C. A. Martínez-Huitle , M. A. Rodrigo , I. Sirés , O. Scialdone , Chem. Rev. 2015, 115, 13362–13407; 26654466 10.1021/acs.chemrev.5b00361

[2b] X. Song , Q. Shi , H. Wang , S. Liu , C. Tai , Z. Bian , Appl. Catal. B 2017, 203, 442–451.

[3a] Y. Li , J. Ma , T. D. Waite , M. R. Hoffmann , Z. Wang , Environ. Sci. Technol. 2021, 55, 10695–10703; 34132087 10.1021/acs.est.1c00264

[3b] B. P. Chaplin , Acc. Chem. Res. 2019, 52, 596–604; 30768240 10.1021/acs.accounts.8b00611

[3c] J. Radjenovic , D. L. Sedlak , Environ. Sci. Technol. 2015, 49, 11292–11302. 26370517 10.1021/acs.est.5b02414

[4a] D. Shuai , B. P. Chaplin , J. R. Shapley , N. P. Menendez , D. C. McCalman , W. F. Schneider , C. J. Werth , Environ. Sci. Technol. 2010, 44, 1773–1779; 20143806 10.1021/es9029842

[4b] Q. Wang , L. Zhou , Q. Chen , M. Mao , W. Jiang , Y. Long , G. Fan , J. Hazard. Mater. 2021, 408, 124456; 33223316 10.1016/j.jhazmat.2020.124456

[4c] X. Yang , L. Liu , M. Zhang , W. Tan , G. Qiu , L. Zheng , J. Hazard. Mater. 2019, 374, 26–34. 30978627 10.1016/j.jhazmat.2019.04.008

[5a] Y. Cai , X. Long , Y.-H. Luo , C. Zhou , B. E. Rittmann , Water Res. 2021, 192, 116841; 33503571 10.1016/j.watres.2021.116841 PMC9753135

[5b] O. El-Sharnouby , H. K. Boparai , J. Herrera , D. M. O′Carroll , Chem. Eng. J. 2018, 342, 281–292.

[6a] B. P. Chaplin , M. Reinhard , W. F. Schneider , C. Schüth , J. R. Shapley , T. J. Strathmann , C. J. Werth , Environ. Sci. Technol. 2012, 46, 3655–3670; 22369144 10.1021/es204087q

[6b] Y. Zhou , G. Zhang , Q. Ji , W. Zhang , J. Zhang , H. Liu , J. Qu , Environ. Sci. Technol. 2019, 53, 11383–11390; 31483614 10.1021/acs.est.9b03111

[6c] J. You , I. Manners , H. Dou , Langmuir 2021, 37, 9089–9097. 34279101 10.1021/acs.langmuir.1c01154

[7a] M. Zhang , D. B. Bacik , C. B. Roberts , D. Zhao , Water Res. 2013, 47, 3706–3715; 23726707 10.1016/j.watres.2013.04.024

[7b] X. Zhou , W. Xu , G. Liu , D. Panda , P. Chen , J. Am. Chem. Soc. 2010, 132, 138–146; 19968305 10.1021/ja904307n

[7c] G. C. Bond , Chem. Soc. Rev. 1991, 20, 441–475.

[8a] J. Ge , J. Lei , R. N. Zare , Nat. Nanotechnol. 2012, 7, 428–432; 22659609 10.1038/nnano.2012.80

[8b] L. van't Hag , S. Handschin , P. M. Gschwend , R. Mezzenga , Adv. Funct. Mater. 2020, 30, 1908458;

[8c] Y. Shen , L. Posavec , S. Bolisetty , F. M. Hilty , G. Nyström , J. Kohlbrecher , M. Hilbe , A. Rossi , J. Baumgartner , M. B. Zimmermann , R. Mezzenga , Nat. Nanotechnol. 2017, 12, 642–647; 28436960 10.1038/nnano.2017.58

[8d] L. Zhang , N. Li , F. Gao , L. Hou , Z. Xu , J. Am. Chem. Soc. 2012, 134, 11326–11329. 22742927 10.1021/ja302959e

[9a] Q. Zhang , S. Bolisetty , Y. Cao , S. Handschin , J. Adamcik , Q. Peng , R. Mezzenga , Angew. Chem. Int. Ed. 2019, 58, 6012–6016; 10.1002/anie.201901596 30791184

[9b] A. Palika , A. Rahimi , S. Bolisetty , S. Handschin , P. Fischer , R. Mezzenga , Nanoscale Adv. 2020, 2, 4665–4670; 10.1039/d0na00189a PMC9419293 36132927

[9c] M. Peydayesh , M. Pauchard , S. Bolisetty , F. Stellacci , R. Mezzenga , Chem. Commun. 2019, 55, 11143–11146. 10.1039/c9cc05337a 31463510

[10a] G. Nyström , L. Roder , M. P. Fernández-Ronco , R. Mezzenga , Adv. Funct. Mater. 2018, 28, 1703609;

[10b] S. S. Bale , P. Asuri , S. S. Karajanagi , J. S. Dordick , R. S. Kane , Adv. Mater. 2007, 19, 3167–3170;

[10c] Y. Pan , W. J. Paschoalino , A. Szuchmacher Blum , J. Mauzeroll , ChemSusChem 2021, 14, 758–791; 33296559 10.1002/cssc.202002532

[10d] Y. Cao , S. Bolisetty , G. Wolfisberg , J. Adamcik , R. Mezzenga , Proc. Natl. Acad. Sci. USA 2019, 116, 4012–4017. 30782823 10.1073/pnas.1819640116 PMC6410827

[11a] M. Peydayesh , S. Bolisetty , T. Mohammadi , R. Mezzenga , Langmuir 2019, 35, 4161–4170; 30811203 10.1021/acs.langmuir.8b04234

[11b] J. Zhao , P. Yang , Adv. Mater. Interfaces 2020, 7, 2001060;

[11c] C. Li , A.-K. Born , T. Schweizer , M. Zenobi-Wong , M. Cerruti , R. Mezzenga , Adv. Mater. 2014, 26, 3207–3212; 24634054 10.1002/adma.201306198

[11d] S. Bolisetty , R. Mezzenga , Nat. Nanotechnol. 2016, 11, 365–371; 26809058 10.1038/nnano.2015.310

[11e] Y. Li , K. Li , X. Wang , M. Cui , P. Ge , J. Zhang , F. Qiu , C. Zhong , Sci. Adv. 2020, 6, eaba1425; 32490204 10.1126/sciadv.aba1425 PMC7239643

[11f] M. Sorriaux , M. Sorieul , Y. Chen , Polymer 2021, 13, 3442; 10.3390/polym13193442 PMC8512863 34641257

[11g] M. Liu , L. D. Jia , Z. X. Zhao , Y. Han , Y. X. Li , Q. M. Peng , Q. R. Zhang , Chem. Eng. J. 2020, 390, 124667.

[12a] Y. Han , Y. Cao , S. Bolisetty , T. Tian , S. Handschin , C. Lu , R. Mezzenga , Small 2020, 16, 2004932; 10.1002/smll.202004932 33090676

[12b] V. Lutz-Bueno , S. Bolisetty , P. Azzari , S. Handschin , R. Mezzenga , Adv. Mater. 2020, 32, 2004941; 10.1002/adma.202004941 33103302

[12c] M. Peydayesh , M. K. Suter , S. Bolisetty , S. Boulos , S. Handschin , L. Nyström , R. Mezzenga , Adv. Mater. 2020, 32, 1907932. 10.1002/adma.201907932 32026524

[13] S. Bolisetty , J. Adamcik , J. Heier , R. Mezzenga , Adv. Funct. Mater. 2012, 22, 3424–3428.

[14] H. Qi , P. Yu , Y. Wang , G. Han , H. Liu , Y. Yi , Y. Li , L. Mao , J. Am. Chem. Soc. 2015, 137, 5260–5263. 25871853 10.1021/ja5131337

[15] Y. Wan , H. Wang , Q. Zhao , M. Klingstedt , O. Terasaki , D. Zhao , Synfacts 2009, 2009, 0697–0697. 10.1021/ja808481g 19275234

[16] P. Gayen , C. Chen , J. T. Abiade , B. P. Chaplin , Environ. Sci. Technol. 2018, 52, 12675–12684. 30239187 10.1021/acs.est.8b04103

[17a] R. Mao , N. Li , H. Lan , X. Zhao , H. Liu , J. Qu , M. Sun , Environ. Sci. Technol. 2016, 50, 3829–3837; 26977556 10.1021/acs.est.5b05006

[17b] R. F. Christman , D. L. Norwood , D. S. Millington , J. D. Johnson , A. A. Stevens , Environ. Sci. Technol. 1983, 17, 625–628; 22288709 10.1021/es00116a012

[17c] Y. Hong , H. Song , T. Karanfil , Water Res. 2013, 47, 1147–1155. 23245540 10.1016/j.watres.2012.11.025

[18a] J. Teng , S. You , F. Ma , X. Chen , N. Ren , Ultrason. Sonochem. 2020, 69, 105248; 32652485 10.1016/j.ultsonch.2020.105248

[18b] J. Teng , G. Liu , J. Liang , S. You , Electrochim. Acta 2020, 331, 135441.

