# Supplementary material for: Amyloid‐Templated Palladium Nanoparticles for Water Purification by Electroreduction
Source: Angew Chem Int Ed Engl. 2022 Jan 31;61(11):e202116634. doi: 10.1002/anie.202116634 (PMC9306645; doi:10.1002/anie.202116634)
Supplement: Supplementary file 1 — Supporting Information [file ANIE-61-0-s001.pdf]

Angewandte Chemie
Eine Zeitschrift der Gesellschaft Deutscher Chemiker

Supporting Information

Amyloid-Templated Palladium Nanoparticles for Water Purification by Electroreduction

J. Teng, M. Peydayesh, J. Lu, J. Zhou, P. Benedek, R. E. Schäublin, S. You, R. Mezzenga**

Author Contributions

J.T. Data curation:Lead; Formal analysis:Lead; Investigation:Lead; Methodology:Lead; Visualization:Lead; Writing – original draft:Lead
M.P. Data curation:Supporting; Formal analysis:Supporting; Writing – original draft:Supporting; Writing – review & editing:Supporting
J.L. Data curation:Supporting; Investigation:Supporting; Writing – original draft:Supporting
J.Z. Data curation:Supporting; Formal analysis:Supporting
P.B. Data curation:Supporting; Formal analysis:Supporting; Methodology:Supporting
R.S. Data curation:Supporting; Formal analysis:Supporting
S.Y. Conceptualization:Supporting; Funding acquisition:Lead; Writing – original draft:Supporting; Writing – review & editing:Supporting

Table of Contents

1. Experimental Procedures
2. Results and Discussion
3. References
4. Author Contributions

Experimental Procedures

Amyloid-templated nano-Pd electrode

Amyloid fibrils preparation

β-lactoglobulin (BLG) was extracted from whey according to previous protocols[1]. BLG monomers (10 g) were dispersed and dissolved in deionized (DI) water (490 mL), resulting in a total volume of 500 mL with a mass fraction of 2 wt%. The pH of the solution was then adjusted to 2 with HCl (1 M). Meanwhile, carbon paper (CP, 20 mm × 20 mm, HCP030N, HESEN, China) was cleaned with DI water, thermally treated (300 ℃, 2 h) for hydrophilicity enhancement, and immersed in the solution. Then, the solution was incubated for 5 h under constant stirring (90 ℃, 200 rpm), during which the monomers were transformed into amyloid fibrils (AFs) through unfolding, hydrolysis, and self-assembly[2]. The fibril formation was terminated by placing the solution in ice. Finally, birefringence was checked in cross-polarized light to confirm the presence of BLG AFs.

Preparation of the high-density nano-Pd hybrid electrode

Various functional groups are available in both AFs and CP, such as -OH and -COOH for CP, and -COOH, $-NH_2$, and -SH for AFs. During the formation of AFs, they can simultaneously bind to CP via different mechanisms including electrostatic interaction, hydrophobic interaction, and hydrogen bonding[2b]. Following the DI water cleaning, we obtained AFs-decorated CP (CP-AFs). Then, CP-AFs was immersed in the solution of Na_2PdCl_4 (0.1 M). Ultrasound was used to promote the dispersion of Pd^{2+} on CP-AFs (4 ℃, 30 min), which was followed by an incubation of 24 h (4 ℃). After the addition of the reducing agent (0.056 M of $NaBH_4$) to the sample dropwise, bright yellow Pd^{2+} on CP-AFs turned black. Following an incubation for another 30 min, nano-Pd loaded CP-AFs (CP-AFs-Pd) was cleaned with DI water to remove the residues, dried in an oven for 12 h (50 ℃), and then stored for further characterization.

In addition, CP was replaced with titanium suboxide reactive electrochemical membrane (TiSO-REM) to synthesize TiSO-REM-AFs-Pd according to the same procedures. Being different from that of CP substrate, the solutions were recirculated by flowing through the porous walls of TiSO-REM to enable sufficient contact with the pore surface. The commercially available carbon paper was used herein due to its good conductivity and functionality for catalyst loading on the surface, making it a suitable substrate for wide applications in lab-scale tests. TiSO was selected due to its porous structure, good conductivity, and stability in electroreduction. Moreover, two commercial Pd-coated carbon paper cathodes (CP-Pd, YanshengBio, China) were also used for control experiments. CP-Pd II had a larger Pd content than CP-Pd I.

Analysis and characterization

Morphology of the electrodes was observed by atomic force microscopy (AFM, MultiMode 8, Bruker, U.S.A.) in soft tapping mode. Prior to AFM measurements, a solution of AFs-Pd was prepared as mentioned above, and an aliquot of the solution was deposited on freshly-cleaved mica sheets. After 2 min, the mica sheets were rinsed by DI water and dried by a gentle flow of compressed air.

Transmission electron microscopy (TEM), scanning TEM (STEM), and high-resolution TEM (HRTEM) images of the electrodes were obtained at accelerating voltages of 80 kV and 200 kV, respectively, with a TEM FEI Talos F200X (U.S.A.) equipped with a field emission gun and large-collection angle energy-dispersive X-ray spectroscopy (EDX) detector (Super-X, Bruker, U.S.A.). AFs were removed from the sample because they would be damaged by the 200-kV accelerating voltage required to gain sufficient spatial resolution to resolve the atomic planes of Pd in HRTEM imaging. The chemical maps were performed at 80 kV to precisely avoid this type of damage, and thus allow visualizing AFs, and at the same time increase the yield in X-rays compared to 200-kV electrons, but then the spatial resolution was not sufficient for resolving the atomic planes of the Pd particles. A diluted solution of AFs-Pd (4 μL) was dripped on a 2-nm carbon-coated copper grid (Quantifoil, Germany) and let for drying in the air for a couple of minutes before TEM measurements.

X-ray diffraction (XRD) analysis was conducted by a powder diffractometer (Siemens D500, Germany) using Cu Kα radiation (λ = 1.5418 Å) with a scan rate of 0.5° s^{-1}. X-ray photoelectron spectroscopy (XPS) patterns were obtained by a spectrometer (PHI-5000 Versaprobe, U.S.A.) using a Mg Kα X-ray source (1253.6 eV photons), and all XPS spectra were corrected by C 1s peak at 284.5 eV.

The structure and properties of the electrodes were characterized by scanning electron microscopy (SEM, LEO 1530, Zeiss, Germany) at an accelerating voltage of 3 kV. Prior to the measurements, small pieces of the electrodes were mounted to aluminium stubs by conductive carbon paste. The elemental composition of the electrodes was determined by energy-dispersive X-ray spectroscopy (EDX, Pegasus system, EDAX, U.S.A.) with a Si(Li) detector connected to SEM. Fourier transform infrared spectroscopy (FTIR, 640-IR, Varian, U.S.A.) equipped with the Imaging Golden Gate Diamond ATR accessory was employed to confirm the coating of AFs on the electrodes. The samples were scanned at room temperature over 4000 cm^{-1} to 400 cm^{-1} with a resolution of 2 cm^{-1}.

The Pd concentration and its loading content on the cathodes were determined by inductively coupled plasma optical emission spectrometry (ICP-OES, Optima 5300 DV, PerkinElmer, U.S.A.). A UV/Vis spectrometer (Cary Series, Agilent, U.S.A.) was used in the range of 250 nm - 700 nm to monitor the catalytic reaction at the absorption peak of 540 nm. The electrochemical properties, including cyclic voltammetry (CV) and electrochemical impedance spectroscopy (EIS), were determined by the electrochemical workstation (CHI760, Chenhua, China) and the workstation (Interface 1000, Gamry, U.S.A.), respectively. CV curves were acquired in the solution of Na_2SO_4 (2 mM) at a scan rate of 1 mV s^{-1}. EIS measurements were performed at open-circuit potential over 100 Hz - 0.01 Hz with an amplitude of 5 mV. Zsimpwin software was used for EIS data-fitting. Electrochemical active surface area (ECSA) was determined according to previous studies[3], using the reference value of capacity per unit area for smooth Pd metals/alloys (23 μF cm^{-2}).

Performance tests

Electrochemical reactor and flow-through experiment

Electroreduction by CP-AFs-Pd was carried out using an H-type reactor, as displayed in Figure 1b. The pre-treated Nafion membrane was vertically placed to separate the anode chamber (200 mL) and the cathode chamber (200 mL). The initial concentration of Cr^{6+} was 1 ppm in the cathode chamber with Na_2SO_4 (2 mM) serving as a supporting electrolyte. The solution pH was adjusted to 5.0 ± 0.1 by H_2SO_4. The reactor was then purged with argon gas and sealed for subsequent experiments. In addition, the argon purge was continued during the whole electroreduction process. The reactor was connected to the electrochemical workstation (CHI760, Chenhua, China), while platinum sheet and Ag/AgCl served as counter and reference electrodes, respectively. Samples were taken from the cathode chamber at predetermined intervals. In the flow-through tests, TiSO-REM-AFs-Pd served as the cathode (Figures 3d,h). The synthetic wastewater was pumped into the hollow porous cathode and then recycled back to the reactor.

The energy consumption during electroreduction tests was calculated according to the previous study[3a]:

$$W\,(\mathrm{kWh\ m^{-3}}) = \frac{U_{\mathrm{cell}} I t}{V} \qquad [Eq.\ \mathrm{S1}]$$

where U_{cell} (V) is the cell voltage, I (A) the current, t (h) the reaction time and V (m^3) the effective volume of the electrolytic cell.

Results and Discussion

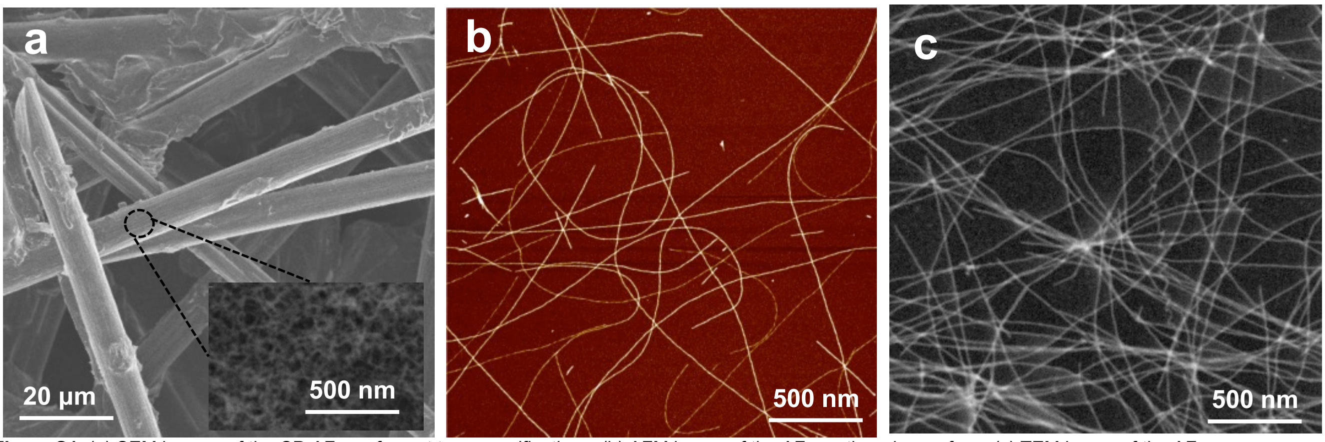

Figure S1. (a) SEM images of the CP-AFs surface at two magnifications. (b) AFM image of the AFs on the mica surface. (c) TEM image of the AFs.

Figures 2d and S1b,c confirmed the expected multistrand twisting of the amyloid protofilaments with a height of approximately 5 nm (Figure 2e).

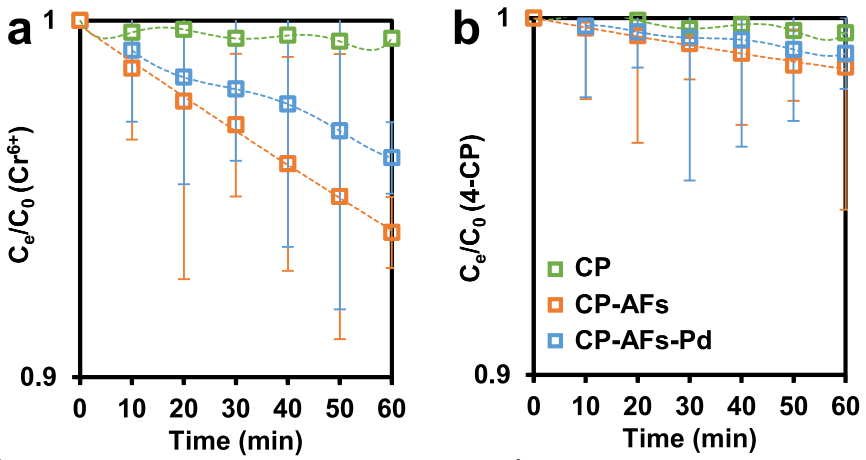

Figure S2. Removal of (a) Cr^{6+} and (b) 4-CP with different cathodes at 0 V vs. SHE. Initial Cr^{6+} concentration = 1 ppm, initial 4-CP concentration = 1 ppm, pH = 6.5, supporting electrolyte concentration = 2 mM.

Only slight removal was found of Cr^{6+} and 4-CP by adsorption on CP, CP-AFs, and CP-AFs-Pd at 0 V vs. SHE (Figure S2).

In addition, the insignificant removal by CP and CP-AFs at -1.2 V vs. SHE was also considered to be the effect of adsorption (Figures 3a,b).

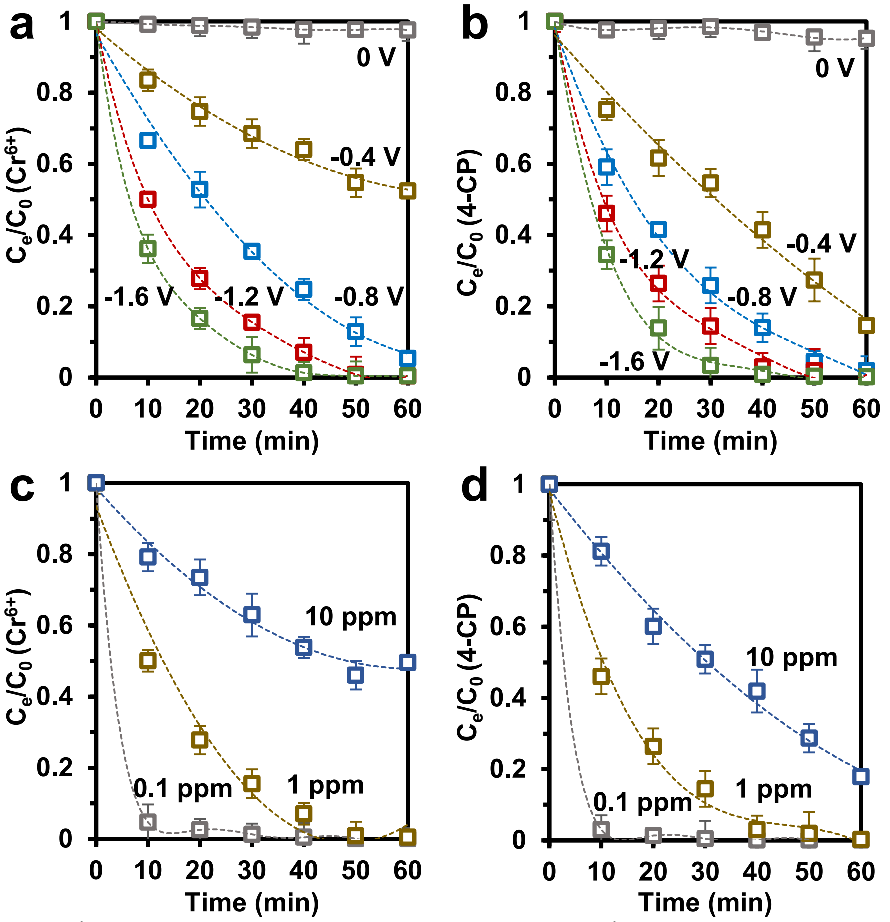

Figure S3. Electroreduction of (a) Cr^{6+} and (b) 4-CP at different potentials. Electroreduction of (c) Cr^{6+} and (d) 4-CP at different initial concentrations. Initial Cr^{6+} concentration = 1 ppm, initial 4-CP concentration = 1 ppm, cathodic potential = -1.2 V vs. SHE, pH = 6.5, supporting electrolyte concentration = 2 mM.

The electroreduction efficiency was positively correlated to applied potential because of the larger number of electrons for Faradaic reaction.

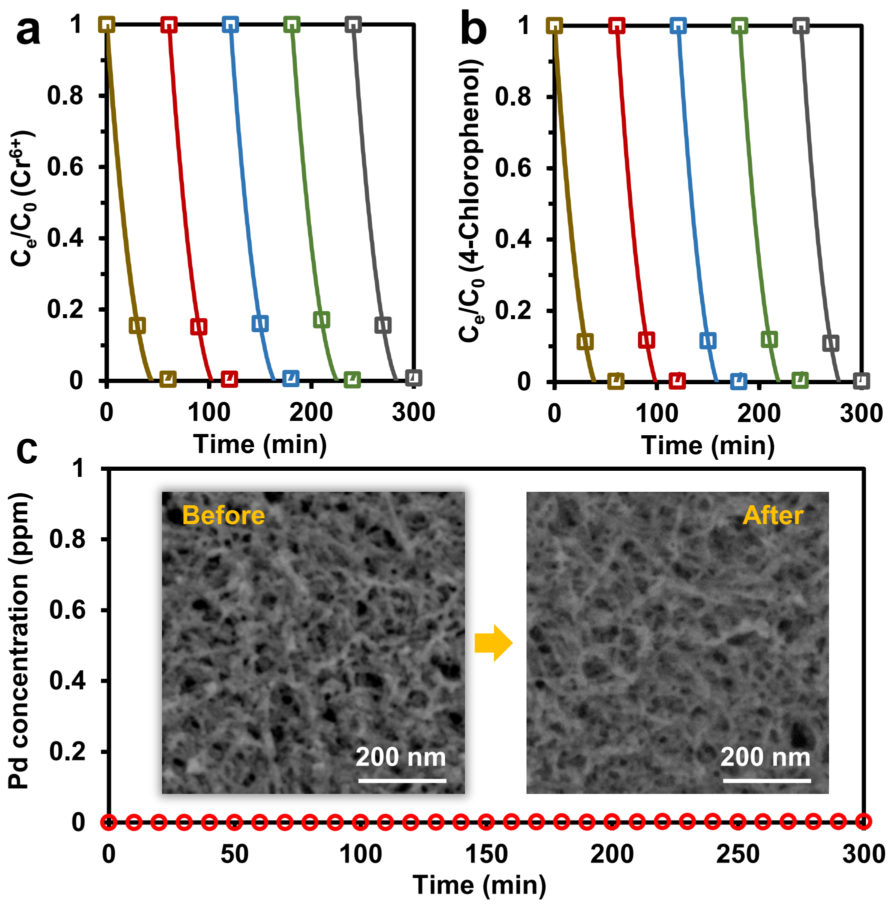

Figure S4. Five-cycle electroreduction of (a) Cr^{6+} and (b) 4-CP by CP-AFs-Pd. (c) Time course of nano-Pd loss on CP-AFs-Pd (Inserted images show the morphology of CP-AFs-Pd before and after the 300-min electroreduction tests). Initial Cr^{6+} concentration = 1 ppm, initial 4-CP concentration = 1 ppm, cathodic potential = -1.2 V vs. SHE, pH = 6.5, supporting electrolyte concentration = 2 mM.

The reusability and the stability of CP-AFs-Pd system were also assessed. After 5 cycles of tests, high reduction efficiency could be sustained with negligile change of surface morphology of the cathode, and insignificant amount of Pd element (<0.003 ppm) was detected in the solution. This indicated the relatively high stability of AFs-Pd on CP (Figure S4).

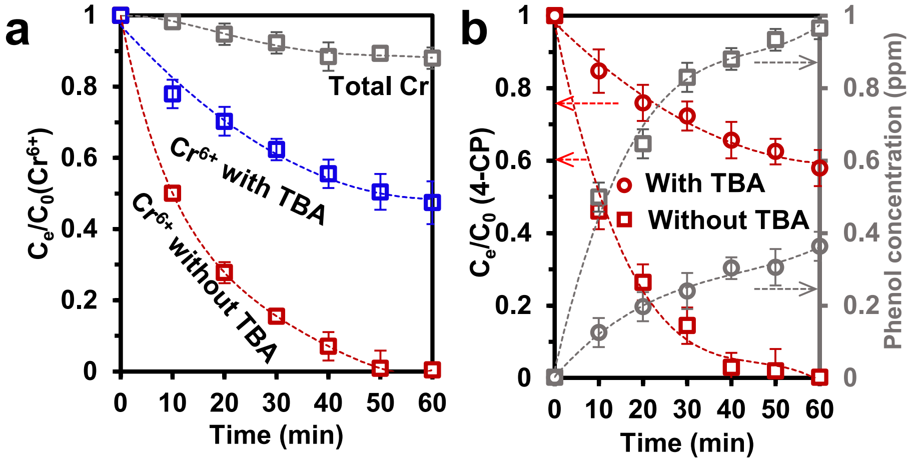

Figure S5. Electroreduction of (a) Cr^{6+} and (b) 4-CP with different levels of TBA. Initial Cr^{6+} concentration = 1 ppm, initial 4-CP concentration = 1 ppm, cathodic potential = -1.2 V vs. SHE, pH = 6.5, supporting electrolyte concentration = 2 mM.

The concentration of total Cr was also decreased by 10%, as AFs tended to adsorb Cr^{3+} after Cr^{6+} reduction (Figure S5a). Phenol was found to constitute the main electroreduction product of 4-CP, with a concentration of approximately 1 ppm (Figure S5b).

To offer further mechanistic insight into H*-mediated electroreduction, tertiary butanol (TBA, 10 mM) was added to scavenge the *in situ* generated H*. As shown in Figure S5, adding TBA led to a 48% and 59% decline in reduction efficiency of Cr^{6+} and 4-CP, respectively, confirming the essential role of H* for electroreduction.

$$H_2O + e^- \leftrightarrow H^* + OH^- \quad \text{(Volmer step)} \qquad \text{[Eq. S2]}$$

$$H_2O + H^* + e^- \leftrightarrow H_2(g) + OH^- \quad \text{(Heyrovsky step)} \qquad \text{[Eq. S3]}$$

$$2H^* \leftrightarrow H_2(g) \quad \text{(Tafel step)} \qquad \text{[Eq. S4]}$$

$$3H^* + Cr^{6+} \rightarrow Cr^{3+} + 3H^+ \qquad \text{[Eq. S5]}$$

$$2H^* + C_6H_5ClO \rightarrow C_6H_6O + H^+ + Cl^- \qquad \text{[Eq. S6]}$$

Prior to H_2 desorption following Heyrovsky or Tafel steps, highly-active H* intermediates generated from water electrolysis on the CP-AFs-Pd electrode served as a robust reducing agent in electroreduction (Eqs. S2-S4). Consequently, electroreduction for Cr^{6+} and 4-CP by H* can be presented in Eqs. S5 and S6.

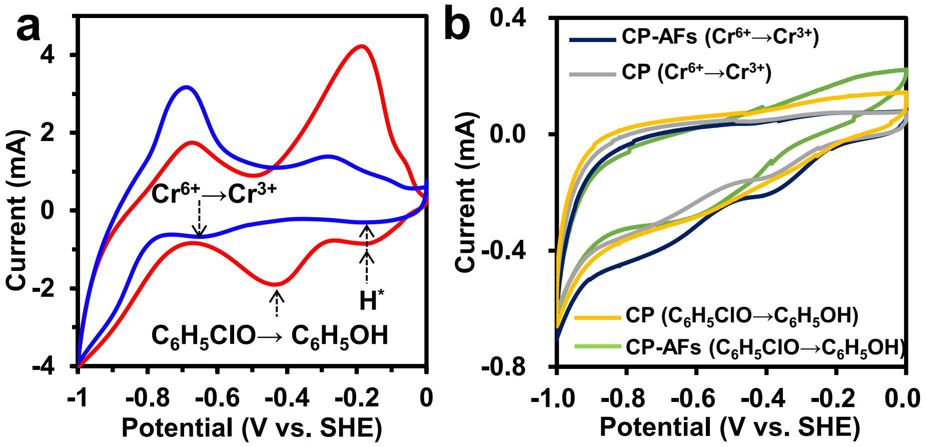

Figure S6. CV curves for (a) CP-AFs-Pd and (b) Pd-free cathodes. Initial Cr^{6+} concentration = 1 ppm, initial 4-CP concentration = 1 ppm, pH = 6.5, supporting electrolyte concentration = 2 mM.

The cyclic voltammetry (CV) tests shown in Figure S6a provide evidence for direct electron transfer by the reduction peaks of Cr^{6+} and 4-CP, and the characteristic peak for H* formation.

Furthermore, Figures S6a,b demonstrate markedly enhanced current by nano-Pd coated on CP-AFs surface.

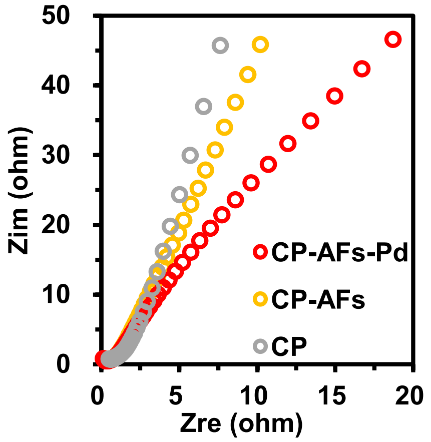

Figure S7. Nyquist plots of the different electrodes in the supporting electrolyte (2 mM).

The CP-AFs-Pd also exhibited lower charge-transfer resistance than Pd-free cathodes (CP and CP-AFs; Figure S7).

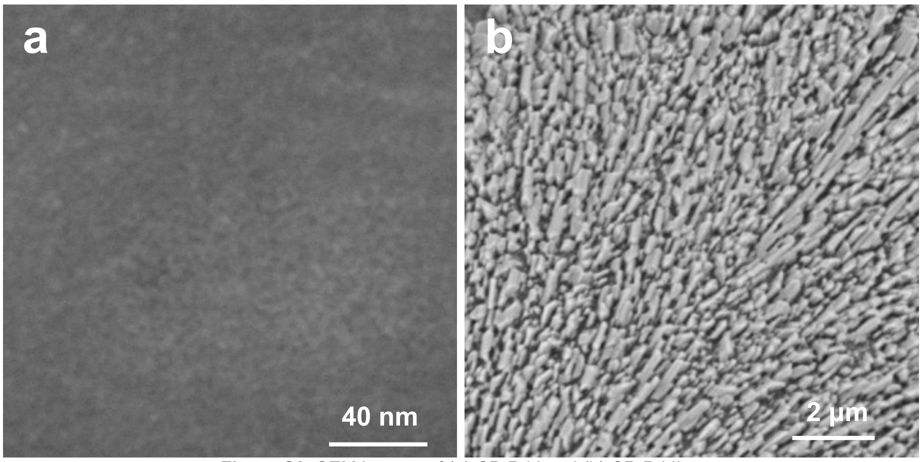

Figure S8. SEM images of (a) CP-Pd I and (b) CP-Pd II.

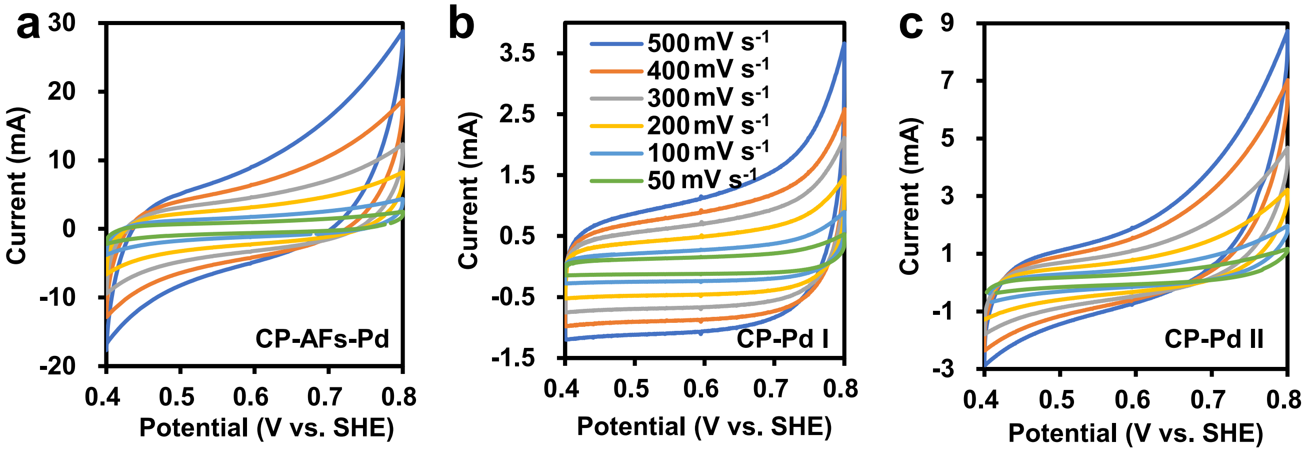

Figure S9. Double-layer current vs. potential at different scan rates obtained in 0.5 M H_2SO_4 supporting electrolyte.

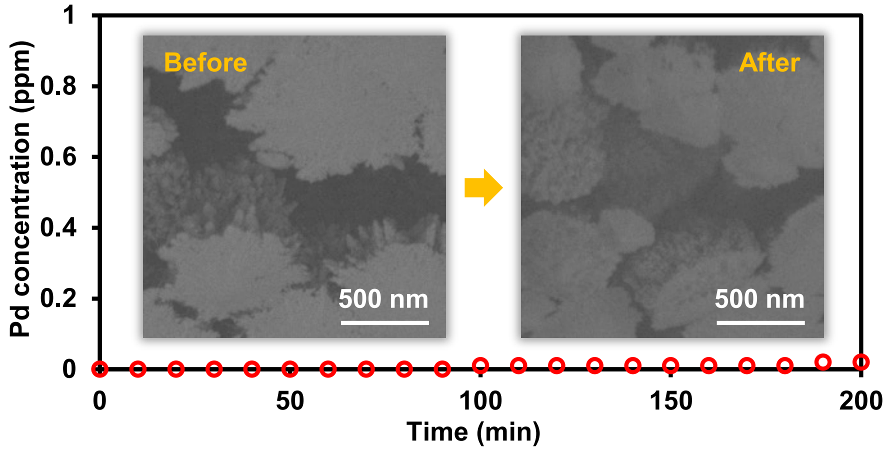

Figure S10. Time course of nano-Pd loss on TiSO-REM-AFs-Pd. Inserted images show the morphology of TiSO-REM-AFs-Pd before and after the 200-min electroreduction tests.

SUPPORTING INFORMATION

Table S1. Summary of electroreduction performance obtained in this work and reported previous studies.

Pollutants	Electrode	Parameters	Time (h)	Removal efficiency	Rate constant (min^{-1})	Ref.
4-CP	Pd/Ppy(SDS)/GC	500 mM H_2SO_4, 1.67 mA cm^{-2}	3	89%	0.011	[4]
	Pd/C gas-diffusion	100 mM Na_2SO_4, 2.44 mA cm^{-2}	1	100%	0.086	[5]
	Pd-Ag/PPY/Ti	50 mM Na_2SO_4, 8 mA	2	94%	0.024	[6]
	Pd/carbon cloth	50 mM NaAc-HAc, 2.8 mA cm^{-2}	15	100%	0.005	[7]
	Pd/RGO/foam-Ni	50 mM Na_2SO_4, 5 mA cm^{-2}	1	100%	0.107	[8]
	Pd/PPy-MWCNTs/Ti	100 mM Na_2SO_4, 1 mA cm^{-2}	2	99.82%	0.042	[9]
	Pd/MWCNTs-B	5 mM Na_2SO_4, 4 mA cm^{-2}	0.5	100%	0.084	[10]
	CP-AFs-Pd	2 mM Na_2SO_4, -1.2 V vs SHE	1	100%	0.095	This work
Cr^{6+}	Graphite felt	20 mL min^{-1}	N/A	94%	0.0719	[11]
	Ti plate	5.6 mM NaCl, 10 mA cm^{-2}	12	96.2%	0.005	[12]
	SiC/XG carbon	500 mM Na_2SO_4, -2.0 V	1.5	96.1%	0.0329	[13]
	Pd film	5 mM Na_2SO_4, -1.0 V vs SCE	4	100%	0.0653	[14]
	Carbon felt	100 mM Na_2SO_4, 40 mA cm^{-2}	4	95.5%	0.0095	[15]
	LGS-PANI	500 L h^{-1} m^{-2}	N/A	100%	0.108	[16]
	MoS_2@SSN	10 g L^{-1} $NaNO_3$, -1.0 V vs Ag/AgCl	1	100%	0.046	[17]
	CP-AFs-Pd	2 mM Na_2SO_4, -1.2 V vs SHE	1	100%	0.096	This work
TCAA	Pd-In/Al_2O_3	2 mM Na_2SO_4, 0.9 mA cm^{-2}	0.5	94%	0.0839	[18]
	Hb/CNT	10 mM KCl, -4.0 V vs SCE	0.67	100%	0.0115	[19]
	Glassy carbon	50 mM Na_2SO_4, -3.0 V vs Ag/AgCl	0.67	100%	0.028	[20]
	Pd/Fe-C	10 mM Na_2SO_4, -1.5 V vs SCE	0.5	96%	0.120	[21]
	Mesh Ag(r)	500 mM Na_2SO_4, 110 mA	3	100%	0.0263	[22]
	GR-Cu foam	2 mM Na_2SO_4, -1.2 V vs SCE	0.67	100%	0.137	[23]
	Pd-Ti/TiO_2NTs	10 mM Na_2SO_4, 100 mA	2	91%	0.019	[24]
	TiSO-REM-AFs-Pd	5 mM Na_2SO_4, -1.2 V vs SHE	0.67	100%	0.113	This work

References

[1] J.-M. Jung, G. Savin, M. Pouzot, C. Schmitt, R. Mezzenga, *Biomacromolecules* **2008**, *9*, 2477-2486.
[2] a) S. Bolisetty, R. Mezzenga, *Nat. Nanotechnol.* **2016**, *11*, 365-371; b) M. Peydayesh, R. Mezzenga, *Nat. Commun.* **2021**, *12*, 3248.
[3] a) S. You, B. Liu, Y. Gao, Y. Wang, C. Y. Tang, Y. Huang, N. Ren, *Electrochim. Acta* **2016**, *214*, 326-335; b) M. Lukaszewski, M. Soszko, A. Czerwinski, *Inter. J. Electrochem. Sci.* **2016**, *11*, 4442-4469; c) L. Fang, Q. Tao, M. Li, L. Liao, D. Chen, Y. Chen, *Chinese J. Chem. Phys.* **2010**, *23*, 543-548.
[4] G. Chen, Z. Wang, T. Yang, D. Huang, D. Xia, *J. Phys. Chem. B* **2006**, *110*, 4863-4868.
[5] H. Wang, J. Wang, *Appl. Catal. B-Environ.* **2007**, *77*, 58-65.
[6] X. Wei, L. Zeng, W. Lu, J. Miao, R. Zhang, M. Zhou, J. Zhang, *Catalysts* **2019**, *9*, 931.
[7] I. F. Cheng, Q. Fernando, N. Korte, *Environ. Sci. Technol.* **1997**, *31*, 1074-1078.
[8] Y. Liu, L. Liu, J. Shan, J. Zhang, *J. Hazard. Mater.* **2015**, *290*, 1-8.
[9] C. Jiang, H. Yu, X. Wang, Y. Lu, X. Luo, *Int. J. Electrochem. Sci* **2017**, *12*, 5208-5219.
[10] X. Shu, Q. Yang, F. Yao, Y. Zhong, W. Ren, F. Chen, J. Sun, Y. Ma, Z. Fu, D. Wang, X. Li, *Chem. Eng. J.* **2019**, *358*, 903-911.
[11] P. Chen, R. Cheng, G. Meng, Z. Ren, J. Xu, P. Song, H. Wang, L. Zhang, *J. Hazard. Mater.* **2021**, *416*, 125768.
[12] F. Yao, M. Jia, Q. Yang, K. Luo, F. Chen, Y. Zhong, L. He, Z. Pi, K. Hou, D. Wang, X. Li, *Chemosphere* **2020**, *260*, 127537.
[13] W. Ma, J. Gao, Z. Chen, J. Hu, G. Xin, Y. Pan, Z. Zhang, D. Tan, *Colloid. Surface. A* **2021**, *610*, 125724.
[14] Y. Hou, H. Liu, X. Zhao, J. Qu, J. P. Chen, *J. Colloid Interf. Sci.* **2012**, *385*, 147-153.
[15] E. P. L. Roberts, H. Yu, *J. Appl. Electrochem.* **2002**, *32*, 1091-1099.
[16] Q. Ji, D. Yu, G. Zhang, H. Lan, H. Liu, J. Qu, *Environ. Sci. Technol.* **2015**, *49*, 13534-13541.
[17] X. Li, C. Wang, F. Liu, Z. Zhang, J. Ali, Q. Long, J. Zhang, *Chem. Eng. J.* **2021**, *404*, 126556.
[18] Y. Liu, R. Mao, Y. Tong, H. Lan, G. Zhang, H. Liu, J. Qu, *Electrochim. Acta* **2017**, *232*, 13-21.
[19] Y.-P. Li, H.-B. Cao, Y. Zhang, *Water Res.* **2007**, *41*, 197-205.
[20] M. D. Esclapez, M. I. Díez-García, V. Sáez, P. Bonete, J. González-García, *Environ. Technol.* **2013**, *34*, 383-393.
[21] A. Li, X. Zhao, Y. Hou, H. Liu, L. Wu, J. Qu, *Appl. Catal. B-Environ.* **2012**, *111-112*, 628-635.
[22] Y. H. Xu, H. Zhang, C. P. Chu, C. A. Ma, *J. Electroanal. Chem.* **2012**, *664*, 39-45.
[23] R. Mao, N. Li, H. Lan, X. Zhao, H. Liu, J. Qu, M. Sun, *Environ. Sci. Technol.* **2016**, *50*, 3829-3837.
[24] W. Xie, S. Yuan, X. Mao, W. Hu, P. Liao, M. Tong, A. N. Alshawabkeh, *Water Res.* **2013**, *47*, 3573-3582.

Author Contributions

Jie Teng: Conceptualization, Methodology, Investigation, Formal Analysis, Writing – Original Draft. **Mohammad Peydayesh:** Methodology, Validation. **Jiandong Lu:** Validation, Resources, Writing – Review & Editing. **Jiangtao Zhou:** Resources. **Peter Benedek:** Resources. **Robin Schaublin:** Resources. **Shijie You:** Conceptualization, Writing – Review & Editing, Funding Acquisition, Project Administration. **Raffaele Mezzenga:** Methodology, Conceptualization, Resources, Supervision.
